# Mitochondrial Function Differences between Tumor Tissue of Human Metastatic and Premetastatic CRC

**DOI:** 10.3390/biology11020293

**Published:** 2022-02-11

**Authors:** Reyniel Hernández-López, Margalida Torrens-Mas, Daniel G. Pons, Maria M. Company, Esther Falcó, Teresa Fernández, Javier M. Ibarra de la Rosa, Pilar Roca, Jordi Oliver, Jorge Sastre-Serra

**Affiliations:** 1 Grupo Multidisciplinar de Oncología Traslacional, Institut Universitari d´Investigació en Ciències de la Salut (IUNICS), Universitat de les Illes Balears, E-07122 Palma de Mallorca, Illes Balears, Spain; r.hernandez@uib.es (R.H.-L.); margalida.torrens@ssib.es (M.T.-M.); d.pons@uib.es (D.G.P.); pilar.roca@uib.es (P.R.); jorge.sastre@uib.es (J.S.-S.); 2 Instituto de Investigación Sanitaria de las Islas Baleares (IdISBa), Hospital Universitario Son Espases, Edificio S, E-07120 Palma de Mallorca, Illes Balears, Spain; mmcompany@hsll.es (M.M.C.); efalco@hsll.es (E.F.); tfernandez@hsll.es (T.F.); jibarra@hsll.es (J.M.I.d.l.R.); 3 Translational Research in Aging and Longevity (TRIAL) Group, Instituto de Investigación Sanitaria de las Islas Baleares (IdISBa), E-07120 Palma de Mallorca, Islas Baleares, Spain; 4 Servicio de Anatomía Patológica, Clinica Rotger, E-07012 Palma de Mallorca, Islas Baleares, Spain; 5 Servicio de Oncología, Hospital Son Llatzer, E-07198 Palma de Mallorca, Illes Balears, Spain; 6 Ciber Fisiopatología Obesidad y Nutrición (CB06/03) Instituto Salud Carlos III, E-28029 Madrid, Spain

**Keywords:** colorectal cancer, metastatic cancer, mitochondrial function, OXPHOS, mtDNA

## Abstract

**Simple Summary:**

Metastasis is an important cause of death from colorectal cancer (CRC). Mitochondria, which are important organelles of cells, play a key role in the metastatic transformation of cancer cells. We aimed to evaluate the adaptations associated with mitochondrial function in tumor tissues from advanced stages of human CRC and whether they could ultimately be used as a therapeutic target in metastatic CRC. We have compared the mitochondrial functionality parameters in tumor tissue samples and the normal adjacent tissue of advanced CRC patients with no radio- or chemotherapy treatment before surgery. Notable differences in mitochondrial functionality were detected between the samples of adjacent tissue versus tumor tissue from metastatic CRC patients. These findings suggest a shift in the mitochondrial function profile occurring in tumor tissue once the metastatic stage has been reached. These changes contribute to promote and maintain the metastatic phenotype, with evidence of mitochondrial function impairment in tumor tissue in the metastatic stage samples.

**Abstract:**

Most colorectal cancer (CRC) patients die as a consequence of metastasis. Mitochondrial dysfunction could enhance cancer development and metastatic progression. We aimed to evaluate the adaptations associated with mitochondrial function in tumor tissues from stages III and IV of human CRC and whether they could ultimately be used as a therapeutic target in metastatic colorectal cancer (mCRC). We analyzed the protein levels by Western blotting and the enzymatic activities of proteins involved in mitochondrial function, as well as the amount of mitochondrial DNA (mtDNA), by real-time PCR, analyzing samples of non-tumor adjacent tissue and tumor tissue from stages III and IV CRC patients without radio- or chemotherapy treatment prior to surgery. Our data indicate that the tumor tissue of pre-metastatic stage III CRC exhibited an oxidant metabolic profile very similar to the samples of non-tumor adjacent tissue of both stages. Notable differences in the protein expression levels of ATPase, IDH2, LDHA, and SIRT1, as well as mtDNA amount, were detected between the samples of non-tumor adjacent tissue and tumor tissue from metastatic CRC patients. These findings suggest a shift in the oxidative metabolic profile that takes place in the tumor tissue once the metastatic stage has been reached. Tumor tissue oxidative metabolism contributes to promote and maintain the metastatic phenotype, with evidence of mitochondrial function impairment in stage IV tumor tissue.

## 1. Introduction

Most patients with colorectal cancer (CRC) die as a consequence of metastasis to other organs, such as the liver and lungs. Among patients with CRC, approximately 50% will eventually develop metastatic colorectal cancer (mCRC) [1]. Over the last decade, treatment for mCRC has had limited success, improving the median survival of treated patients from approximately 6 months to 2 years, although few patients with metastatic disease are cured. In fact, many mCRC acquire drug resistance during treatment, while others present with intrinsic chemoresistance [2,3].

Cancer development and metastatic progression could be influenced by mitochondrial dysfunction, and energy metabolism has been implicated in tumor drug resistance [4,5]. Consequently, there is a need for a better understanding of the mechanistic links of energy metabolism in the advanced stages of CRC that could lead to an alternative approach for treatments to overcome drug resistance [2].

Energy metabolism is basic and necessary to ensure cell growth and proliferation. Cancer cells face a metabolic challenge, due to their rapid cell growth and replicative immortality, which force them to adapt their whole metabolism to meet this impressive energy demand [6]. Normal differentiated cells rely primarily on oxidative phosphorylation (OXPHOS). However, in most cancer cells, a metabolic change predominates from OXPHOS towards aerobic glycolysis, a switch that is known as "the Warburg effect" [7] and has been widely observed [8]. In this regard, altered energy metabolism has recently emerged as a hallmark of cancer [9]. In several types of tumors, glycolysis is widespread as the main and, in some cases, the exclusive source of adenosine 5′-triphosphate (ATP). However, some types of cancers do not promote intense glycolysis and instead depend on OXPHOS for ATP production [10]. Although the generation of ATP through aerobic glycolysis is an inefficient process, it confers a certain advantage to cancer cells, allowing them to survive in a harsh tumor environment. Indeed, glycolytic intermediates are diverted from glycolysis to various biosynthetic pathways through which the cell obtains macromolecules to support the generation of new cells, promoting an increase in the tumor biomass rather than efficient ATP production [6,11].

In the current study, we aimed to evaluate the changes associated with the mitochondrial function in tumor tissues of stage III and stage IV human CRC to describe potential changes that could be ultimately used to design therapeutic strategies in mCRC. Protein levels and enzymatic activities of proteins involved in energy metabolism and mitochondrial function were analyzed, as well as the amount of mitochondrial DNA (mtDNA), comparing non-tumoral adjacent tissue and tumor tissue of stage III and stage IV CRC patients.

## 2. Materials and Methods

### 2.1. Patient Selection and Tissue Samples

A total of 23 patients with advanced CRC were selected to be included in this study. This was conducted in accordance with the “World Medical Association Declaration of Helsinki”, and all patients gave their informed consent prior to their inclusion in the study. Patients did not receive any radio or chemotherapy treatment prior to surgery and did not present comorbidity. The mean age of the male subjects was 71.3 years (SD: 8.7, range: 56–84) and 71.0 years for females (SD: 8.8, range: 55–81). The tumor samples were histologically identified as adenocarcinomas and collected by the Biobank of the Hospital of Son Llàtzer. Eleven samples were analyzed for each stage, and both tumor tissue and normal mucosa (non-tumor adjacent tissue) were taken from each patient. All tumor tissues were classified according to the TNM system by an expert pathologist. Detailed information of patients and samples can be found in [12].

### 2.2. Tissue Sample Preparation

Tissue samples were homogenized as previously described [12]. The Bradford method was used for protein quantification. These samples were used to analyze the protein levels of sirtuin 1 (SIRT1), peroxisome proliferator-activated receptor gamma coactivator-1alpha (PGC1α), mitochondrial transcription factor A (TFAM), lactate dehydrogenase A (LDHA), pyruvate dehydrogenase E1 component subunit alpha (PDH), isocitrate dehydrogenase 2 (IDH2), and OXPHOS complexes and for the analysis of the IDH2, cytochrome c oxidase (COX), and ATP synthase (ATPase) enzymatic activities. 

### 2.3. Western Blot Analysis

Protein levels of OXPHOS complexes, SIRT1, PGC1α, TFAM, LDHA, PDH, and IDH2 were analyzed by the Western blotting technique, loading equal amounts of each sample: 20 μg for SIRT1 and IDH2; 30 μg for the PGC1α, LDHA, PDH, and OXPHOS complexes; and 40 μg for TFAM. The samples were loaded in 12% precast gels as previously described [12], transferred to a 0.2 µm nitrocellulose membrane using the Trans-Blot Turbo Transfer System (Bio-Rad Laboratories, CA, USA). Each membrane was subjected to a Ponceau staining to make sure the transfer was conducted successfully. Following 1h of blocking with 5% non-fat powdered milk, primary antibodies were added against the OXPHOS complexes (Abcam, Bristol, UK; Cat# ab110411, RRID:AB_2756818); SIRT1 (Cell Signaling Technology, Danvers, MA; Cat# 8469, RRID:AB_10999470); PGC1α (Abcam Cat# ab54481, RRID:AB_881987); TFAM (Cell Signaling Technology, Danvers, MA, USA; Cat# 7495, RRID:AB_10841294); LDHA (Cell Signaling Technology, Danvers, MA, USA; Cat# 2012, RRID:AB_2137173); PDH-E1α (Santa Cruz Biotechnology, Santa Cruz, CA, USA; Cat# sc-377092, RRID:AB_2716767), and IDH2 (Cell Signaling Technology, Danvers, MA; Cat# 56439, RRID:AB_2799511). Then, the membranes were washed and incubated for 1h at room temperature with secondary antibodies: anti-rabbit (Sigma-Aldrich, St Louis, MO, USA; Cat# A9169, RRID:AB_258434) or anti-mouse (Sigma-Aldrich, St Louis, MO, USA; Cat# A9044, RRID:AB_258431). A Chemidoc XRS densitometer (Bio-Rad Laboratories, Hercules, CA, USA) was used to obtain the chemiluminiscence signal. Image analysis was conducted with Quantity One Software (Bio-Rad Laboratories, Hercules, CA, USA; Quantity One 1-D Analysis Software, RRID:SCR_014280). The TGX Stain-Free system from BioRad was used and a Ponceau staining was used as a loading control. For more details and to observe representative images, check Supplementary Method S1 and Figure S1. Furthermore, in each gel, two samples consisting of a mix of the four types of samples used (stage III non-tumor and tumor tissue and stage IV non-tumor and tumor tissue) were loaded, and the results were normalized to this mean value in order to compare between gels, as conducted before [12].

### 2.4. IDH Activity

The IDH activity was determined in each homogenized sample using a spectrophotometric assay [13] with the following modifications. A total volume of 5 µL of homogenate was incubated in assay buffer (0.1 M TAE, pH 7.3) in the presence of 100 mM MnCl_2_ and 13 mM NADP^+^. Then, 67 mM DL-isocitrate was added. The increase in absorbance by the reduction of NADP^+^ was followed for 25 min at 339 nm and 37 °C. The slope was directly proportional to the activity of IDH.

### 2.5. COX Activity

The COX activity was determined in each homogenized sample using a spectrophotometric assay [14] with the following modifications. A total volume of 25 µL of homogenate was incubated in 0.1 M NaPO_4_H_2_, pH 7.0, with 2 µg/mL catalase and 5 mM substrate DAB (3,3’-diaminebenzidine-tetrachloride). After incubation, 100 µM reduced cytochrome c was added. The increase in absorbance by the oxidation of DAB was followed for 30 min at 450 nm and 37 °C. The generated slope was directly proportional to the activity of COX.

### 2.6. ATPase Activity

ATPase activity was determined in each homogenized sample using a spectrophotometric assay [15] with the following modifications. A total volume of 5 µL of homogenate was incubated in the assay buffer (0.33 M sucrose, 6.3 mM MgSO4, 63.33 mM HEPES, and 0.442 mM NADH, pH 8.0) with 0.1 M Phospho(enol)pyruvic acid, 10 µg/mL pyruvate kinase, 10 µg/mL l-lactic dehydrogenase, and 2 µg/mL antimycin; following incubation, 0.1 M ATP was added. The decrease in absorbance by the oxidation of NADH was followed for 15 min at 340 nm and 37 °C. The generated slope was inversely proportional to the activity of ATPase.

### 2.7. mtDNA Quantification

DNA of each tissue sample was isolated from slides of 10 µm using a QIAamp® FFPE tissue kit (Qiagen, Hilden, Germany) following the manufacturer's instructions. The total DNA was quantified with a spectrophotometer at 260 nm. Next, 5 ng of the total DNA was amplified by real-time PCR using specific primers for 18S (forward 5′-GGA CAC GGA CAG GAT TGA CA-3′ and reverse 5′-ACC CAC GGA ATC GAG AAA GA-3′ for 18S) and the NADH dehydrogenase subunit 4 (forward 5′-CGT GAC TCC TAC CCC TCA CA-3′ and reverse 5′-ATC GGG TGA TGA TAG CCA AG-3′) on a LightCycler 480 System II (Basel, Switzerland, UChicago IGSB Next Generation Sequencing Core, RRID: SCR_011063) with 7.5 μL of Lightcycler^®^ 480 SYBR Green I Master (Basel, Switzerland), 0.5 μM of each set of primers, and 2.5 μL of the cDNA template. The PCR program was 95 °C for 5 min, and 45 cycles of 95 °C for 10 s, 60 °C for 10 s, and 72 °C for 12 s. A negative control was run in each assay. A ratio between the resulting PCR products (NADH dehydrogenase subunit 4/18S) was analyzed to evaluate the amount of mtDNA. 

### 2.8. Statistics

The protein levels are represented graphically by a boxplot. The enzymatic activities and mtDNA quantification data are presented as the means ± the standard deviations (SD). The statistical analyses were conducted with the Statistical Program for the Social Sciences V21.0 (SPSS, Armonk, NY: IBM Corp, RRID:SCR_002865). Analyses of the differences between experimental groups were conducted with ANOVA, two-way and one-way analysis, with the statistical significance set at *p* < 0.05.

## 3. Results

### 3.1. OXPHOS Complex Protein Expression Levels

**Figure 1:**
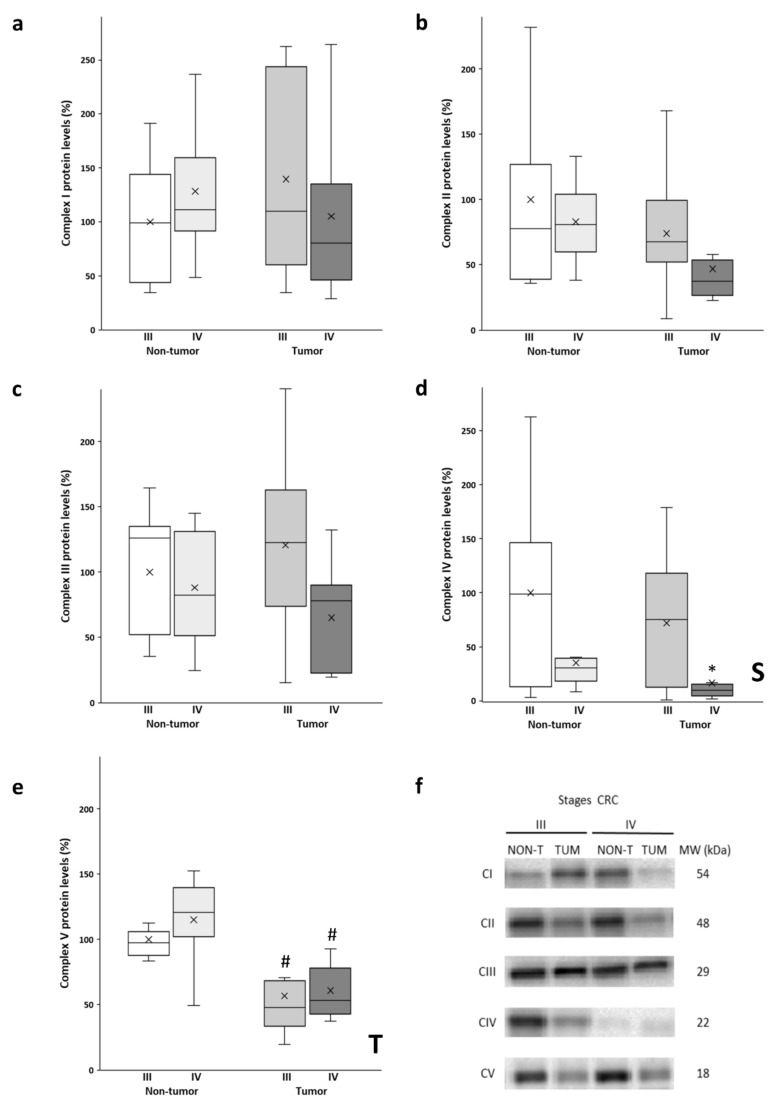
Boxplot of the protein levels of the OXPHOS complexes: Complex I (**a**); Complex II (**b**); Complex III (**c**); Complex IV (**d**) and Complex V (**e**). The X in each boxplot represents the mean. ANOVA analysis: two-way (n=11 per group): S, stage effect; T, tissue effect; one-way (*n* = 11 per group): * difference between stage III and stage IV, # difference between non-tumor and tumor tissue; (**f**): Representative bands of the OXPHOS complexes; NON-T, non-tumor adjacent tissue; TUM, tumor tissue.

The protein levels of the OXPHOS complexes (Figure 1a–e) were determined in both the tumor tissue and adjacent non-tumor tissue. As can be observed in Figure 1a,c, no significant differences were found in Complex I and Complex III levels between the experimental groups. Although the stage III tumor tissue of had the highest protein levels of both complexes, no significant differences were observed, as these tissue samples also had a greater data dispersion. Conversely, lower levels of both complexes were observed in the stage IV tumors when compared to their non-tumor counterparts. In contrast, no significant differences were found in the levels of Complex II (Figure 1b). In addition, stage IV tumors showed lower levels of this complex compared to all the other analyzed groups.

A similar tendency in the levels of Complex V (Figure 1e) was obtained, as the tumor samples of both stages III and IV showed significantly lower protein levels compared to their corresponding non-tumor adjacent tissue. Furthermore, non-tumor adjacent tissue of stage IV showed the highest protein levels for Complex V.

As noted in Figure 1d, significantly lower levels of Complex IV were observed in both the adjacent and tumor tissue of stage IV with respect to stage III. In addition, the tumor tissue of stage IV showed much lower protein levels compared to stage III.

### 3.2. Metabolic Enzyme Levels

**Figure 2:**
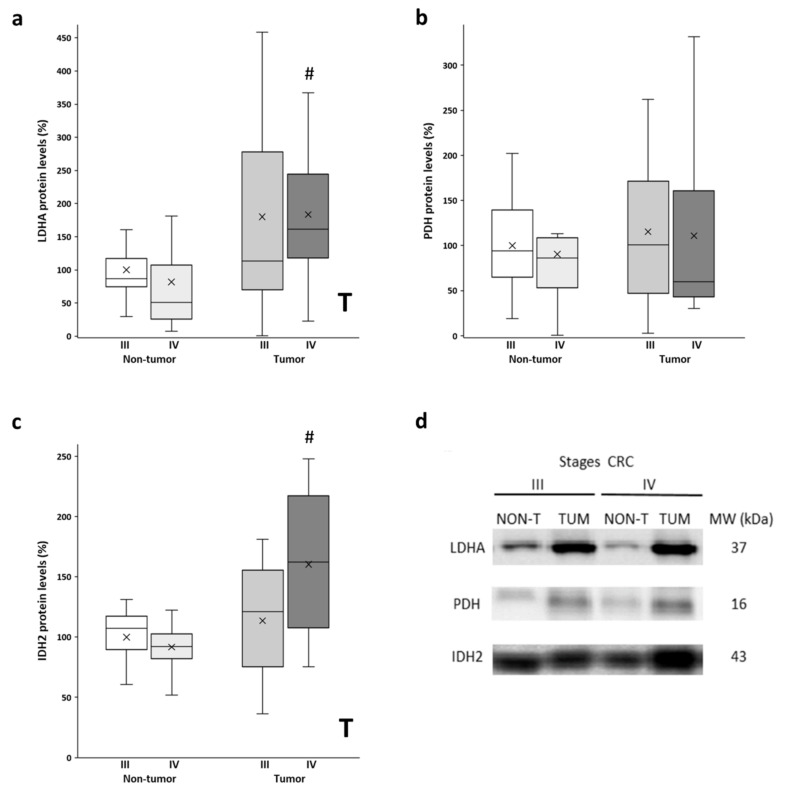
Boxplot of the protein levels of LDHA (**a**), PDH (**b**), and IDH2 (**c**). The X in each boxplot represents the mean. ANOVA analysis: two-way (*n* = 11 per group): T, tissue effect; one-way (*n* = 11 per group): # difference between non-tumor adjacent and tumor tissue. (**d**) Representative bands of LDHA, PDH, and IDH2; NON-T, non-tumor adjacent tissue; TUM, tumor tissue.

After analyzing the mitochondrial function with the levels of OXPHOS, we also checked the metabolic enzymes LDHA and PDH. LDHA and PDH convert pyruvate to lactate and acetyl-CoA, respectively. Thus, LDHA is an indicator of the Warburg effect, while PDH regulates the entry of fuel to the mitochondria. Higher protein levels of LDHA (Figure 2a) were observed in the tumor samples compared to the non-tumor samples. The stage III tumor tissue showed a much greater data dispersion. Furthermore, similar levels of PDH protein expression (Figure 2b) were found in the adjacent and tumor tissues in both stages. Furthermore, we also analyzed the levels of IDH2, a mitochondrial enzyme that participates in the Krebs cycle. On the other hand, significant differences, but only for stage IV, were found for protein levels of IDH2 (Figure 2c), as the tumor showed higher protein levels compared to the non-tumor adjacent tissue. In addition, although not statistically significant, stage IV tumor samples showed higher IDH2 levels compared to stage III tumors.

### 3.3. SIRT1, PGC1α, and TFAM Levels

**Figure 3:**
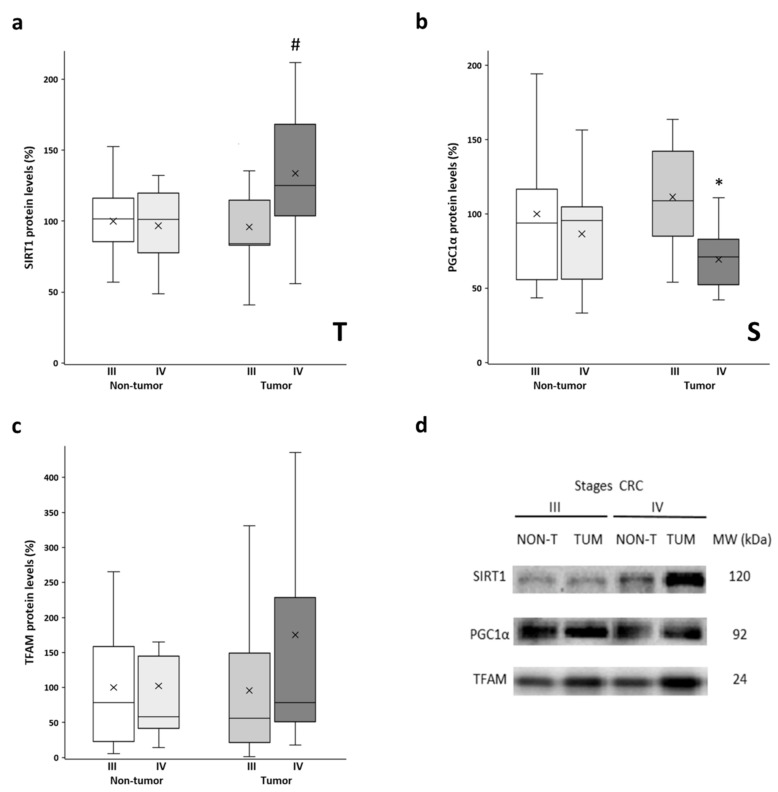
Boxplot of the protein levels of SIRT1 (**a**), PGC1α (**b**), and TFAM (**c**). The X in each boxplot represents the mean. ANOVA analysis: two-way (*n* = 11 per group): T, tissue effect; S, stage effect; one-way (*n* = 11 per group): * differences between stage III and stage IV, # differences between non-tumor adjacent tissue and tumor tissue; (**d**) Representative bands of SIRT1, PGC1α, and TFAM; NON-T, non-tumor adjacent tissue; TUM, tumor tissue.

The protein levels of SIRT1, PGC1α, and TFAM were determined in both the tumor tissue and non-tumor tissue samples. These proteins are the main regulators of mitochondrial biogenesis, with PGC1α being the master regulator of this process. SIRT1 is responsible for PGC1α deacetylation and activation, while TFAM is a downstream target of PGC1α that regulates mitochondrial DNA replication and transcription. Similar levels of SIRT1 were observed in non-tumor tissues from both stages and stage III tumors (Figure 3a). However, the stage IV tumor tissue showed significantly higher SIRT1 levels compared to their non-tumor counterpart. No differences between adjacent and tumor tissues were found for PGC1α levels (Figure 3b). However, both tissues from stage III showed significantly higher PGC1α levels than those from stage IV. Very similar levels of TFAM (Figure 3c) were observed when comparing non-tumor tissues and stage III tumors. Nonetheless, the stage IV tumor samples showed higher levels of TFAM, although these samples had a greater data dispersion, and the differences did not reach statistical significance. Furthermore, some of these results were replicated in vitro by comparing a primary CRC cell line and a metastatic CRC cell line (Supplementary Figure S2; check Supplementary Method S2 for details on cell culture handling), as well as by querying public databases of gene expression data (Supplementary Figure S3; check Supplementary Method S3 for details on data analysis).

### 3.4. IDH2, COX, and ATPase Enzymatic Activities

**Figure 4:**
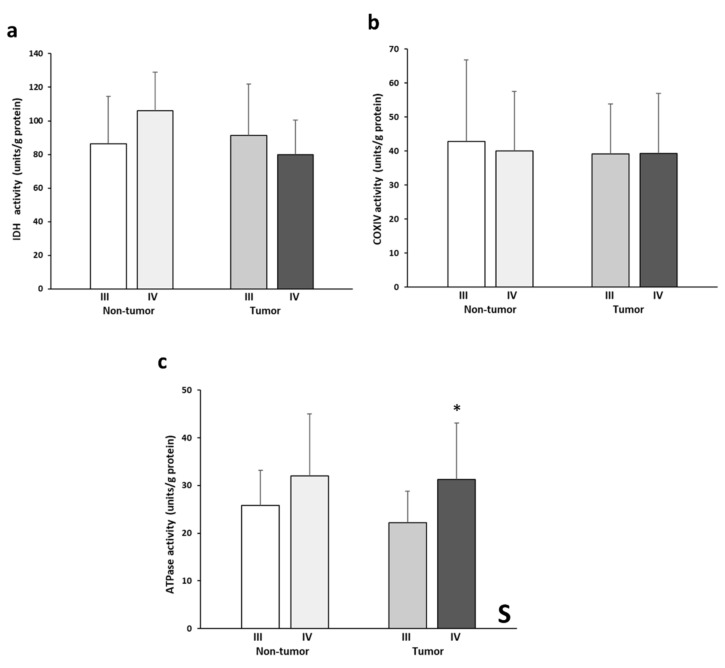
Enzymatic activities of IDH (**a**), COX (**b**), and ATPase (**c**). The data are presented as means ± SD. ANOVA analysis: two-way (*n* = 11 per group): S, stage effect; one-way (*n* = 11 per group): * difference between stage III and stage IV.

Figure 4 shows the levels of IDH2, COX, and ATPase enzymatic activities. IDH2 activity levels (Figure 4a) almost presented a significant interactive effect between tissue and stage (*p*= 0.054). This pattern is reflected by the higher enzymatic activity in the non-tumor stage IV tissue and, conversely, by the lower activity in the stage III tumor tissue. No significant differences in COX activity (Figure 4b) were found between the experimental groups analyzed. The tumor tissues of both stages showed very similar COX activity values. On the other hand, significantly greater ATPase activity (Figure 4c) was found in the stage IV samples compared to stage III. Finally, stage IV tumor tissue showed higher values of ATPase activity than the stage III tumor tissue.

### 3.5. mtDNA Quantification

The number of copies of the mtDNA in the tumor and non-tumor tissue was evaluated. The results are reported in Table 1. As noted, the mtDNA copy number presented a significant tissue effect that was reflected by a significantly higher mtDNA copy number in the tumor tissue of both stages compared to their corresponding non-tumor adjacent tissue. Moreover, both tumor tissue samples of advanced stage of CRC showed very similar values of the mtDNA copy number.

## 4. Discussion

In the current study, we evaluated the adaptations associated with energy metabolism in tissue samples from the advanced stages of human CRC to gain a better understanding of mitochondrial function in human mCRC that could lead to the design of therapeutic strategies in advanced-stage (III and IV) CRC. Our findings suggest that the tumor tissue in a pre-metastatic stage III of CRC presents an oxidant metabolic profile very similar to that of its non-tumor adjacent tissue, whereas in stage IV of CRC, the oxidative metabolism contributes to maintaining the metastatic phenotype, with evidence of mitochondrial function impairment.

Proper mitochondrial function is crucial for energy production through the synthesis of ATP by the Krebs cycle and the OXPHOS complexes. An important decrease in OXPHOS complex IV was observed in the stage IV tumor tissue compared to stage III. Complexes I-III showed only a tendency to decrease in stage IV. Interestingly, the same pattern of OXPHOS protein levels was observed in the non-tumor adjacent tissue, except for complex I, suggesting that mitochondrial function is also affected in this type of tissue. Conversely, the tumor tissue of both stages showed lower levels of complex V compared to the non-tumor adjacent tissue. Similar results to the OXPHOS protein expression levels observed in stage III CRC were reported in tumor tissue and its corresponding adjacent control tissue in the intestinal-type gastric carcinoma [16]. Reactive oxygen species (ROS) production is tightly regulated in tumor cells, as ROS are responsible for cancer survival and progression, metabolic reprogramming, immune evasion, and metastasis. In fact, it has been reported that the activation of matrix metalloproteinases, which are key enzymes for the epithelial-mesenchymal transition and the initiation of metastasis, is ROS-dependent. Complexes I and III of the mitochondrial respiratory chain are the primary generators of ROS within mitochondria, accounting for 80% of the superoxide generated by the ETC [17]. Thus, it could be argued that the reduction in these two complexes could reduce the levels of ROS production in stage IV tumors. However, it must be noted that not only the levels of OXPHOS complexes but also their activity, their assembly into supercomplexes, and the flow of electrons through the whole chain must be considered [18]. In this regard, we have previously reported in the same samples an increase in oxidized glutathione and in lipid peroxidation in stage IV tumor tissue, suggesting that there still exists a redox imbalance that cannot be palliated. On the other hand, a great increase in ROS production and oxidative stress could be detrimental for the cell, as it may trigger cell death [19]. Therefore, the reduction in OXPHOS complexes in stage IV tumor tissue could be limiting ROS production to prevent ROS-mediated cell death.

Despite the lower protein levels of the OXPHOS complexes in stage IV, tumor tissue it is possible that mitochondrial function is not entirely compromised, as the enzymatic activities of both COX and ATPase were preserved. This apparent contradiction has been previously reported in other studies using cell lines and rats, emphasizing the importance to analyze both the levels and enzymatic activities [20,21,22,23,24,25,26,27]. It must be noted that most of the COX subunits are subjected to reversible modification by phosphorylation. Some phosphorylation sites may reduce its activity, while others increase COX activity instead and reduce its inhibition by ATP [28]. This reversible modification allows cells to rapidly respond to changes in the microenvironment and regulate both the mitochondrial membrane potential and reactive oxygen species production [29]. Thus, it is possible that COX posttranslational modifications could be compensating for the reduction observed in its levels. On the other hand, some reports show that some stimuli, such as hypoxia, can induce the expression of some specific subunit isoforms while targeting others for degradation. In this case, it is also possible that the tumor is responding to several signals that cause the change in the Complex IV subunit isoforms and favor its activity. Furthermore, COX is the most regulated enzyme of the electron transport chain, as its activity ultimately controls the efficiency of the whole chain [30]. Thus, our results could indicate that COX activity may be promoted in both the tumor and its surrounding environment in the metastatic stage, with a potential mechanism that would increase the efficiency of the ATPase enzymatic activity, which was in fact increased in the stage IV tumoral tissue. 

Together, these adaptations could maintain the energetic functionality of these metastatic cells. In fact, a previous study reported an upregulation of the functional capacity of OXPHOS and the respiration rate in human CRC samples and their corresponding non-tumor adjacent tissue compared to normal tissue, although it does not differentiate between the stages of CRC tissue [31]. In line with this, the flow of substrates in the mitochondria towards oxidative metabolism would be guaranteed, due to the conserved protein levels in stage III and IV tumor tissue of a critical gate enzyme, PDH. In contrast, the high levels of LDHA observed in the tumor tissue of both advanced stages of CRC would suggest a clear Warburg effect, which indicates that aerobic glycolysis would also be supplying energy to promote and maintain the metastatic stage in the human CRC. According to these results, it was reported that a high expression of glycolytic genes in tumor tissue is associated with a poor prognosis of patients with mCRC [32].

On the other hand, mutations in IDH2 have been observed in various human cancers, including CRC [33]; these mutations have been associated with a loss of IDH2 native enzymatic activity [34]. Our results showed higher levels of IDH2 in tumor tissue of mCRC, which could be an attempt to compensate for its lower native enzymatic activity compared with its non-tumor counterpart. In fact, the enzymatic activity was conserved in all tissues, suggesting that both aerobic glycolysis and the Krebs cycle are activated in the advanced stages of CRC. However, it is important to note that the enzymatic activity reported here does not differentiate between the IDH isoforms. On the other hand, IDH2 also participates in the regulation of oxidative stress, which could explain why its levels were upregulated. Our previous study reported that the non-tumor adjacent and tumor tissue of a mCRC stage presents higher protein levels of antioxidant enzymes in response to higher levels of oxidative stress [12].

The maintenance of both the appropriate structure and function of mitochondria depends on mitochondrial biogenesis, which implies the proliferation and differentiation of mitochondria and constitutes an adaptive mechanism in response to changes in energy demand [35]. SIRT1 acts through direct deacetylation, leading to the activation of its substrates, such as the transcriptional coactivator PGC1α [36], the master regulator of mitochondrial biogenesis, oxidative energy metabolism, adaptive thermogenesis, and fatty acid metabolism [37,38]. We found signs of mitochondrial biogenesis induction in the pre-metastatic stage due to the higher protein levels of PGC1α observed in stage III compared to stage IV. Although a previous study showed lower mRNA levels of PGC1α in CRC tumors compared to normal mucosa [39], we only observed this reduction in mCRC. In contrast, the tumor tissue of mCRC displayed the highest protein levels of SIRT1. In previous studies, we observed a similar protein expression pattern for SIRT1 and PGC1α in a metastatic colon cancer cell line [23]. Despite the downregulation of PGC1α observed in the stage IV tissue, the higher levels of SIRT1 could compensate for this reduction and activate PGC1α by deacetylation to activate mitochondrial biogenesis and rescue OXPHOS levels, although the acetylation status of PGC1α was not checked in this study. This would be in line with a study that showed an increase in the protein levels of the OXPHOS complexes that are SIRT1/PGC1α-axis-dependent in human colonosphere cultures derived from mCRC liver patient tissues [40]. 

In this regard, we observed increased levels of TFAM in stage IV tumor tissue, although it was not statistically significant. TFAM is activated through PGC1α via nuclear respiration factors 1/2 to promote mitochondrial biogenesis and function in response to different external stimuli [41]. TFAM is the most important activator of the mitochondrial genome, as it regulates mitochondrial genome replication and transcription and plays a critical role in maintaining the structure and copy number of the mitochondrial genome [42,43]. The tumor tissues of both stages of CRC showed a differential pattern in the levels of TFAM and PGC1α. Similar results were reported in a subtype of epithelial ovarian carcinoma [44]. These results suggest that mitochondrial function is promoted in tumor tissue. TFAM levels are regulated by other factors, independent of PGC1α. It has been reported that oxidative stress may increase the phosphorylation of the nuclear respiratory factor 1 (NRF1), which then binds to the TFAM promoter and activates its transcription [45]. On the other hand, the degradation of TFAM is also an important point of regulation. When TFAM is phosphorylated, it detaches from mtDNA and is degraded by mitochondrial proteases [46]. Other regulators of TFAM include the tumor suppressor p53 [47,48], although we have previously shown that p53 is reduced in tumor tissue of both stages [12] and, therefore, do not believe that p53 regulates TFAM levels in these samples. Another described mechanism for TFAM regulation is through TGFβ signaling, although this mechanism is yet to be proven in mammals [49]. Finally, several microRNAs have been recently reported to regulate the levels of TFAM by interfering with the translation of its mRNA [50,51,52].

We have also replicated some of these results in in vitro experiments using the HT-29 and SW620 cell lines. However, the data obtained must be carefully considered. First, the TNM stage of the HT-29 cell line is not known, and here we have compared specifically stages III and IV of CRC tissues. Furthermore, it is not possible to replicate the non-tumor adjacent tissue from the same individual in vitro, and we have shown here and in other studies [12,53] that this type of tissue is highly influenced by the presence of the tumor and reflects the different stages of CRC. We have also analyzed data from public databases to further examine the influence of proteins related to mitochondrial function in CRC. These data additionally support the findings reported in this study. 

Finally, we found that the tumor tissues of both stages displayed a greater mtDNA copy number with respect to their non-tumor adjacent tissue, which indicates a change in the mitochondrial content in response to mitochondrial biogenesis promotion. Similar results were obtained in stages III and IV of human CRC, where tumor tissue displays an increase in the mtDNA copy number compared with its non-tumor adjacent tissue [54]. Alterations in the mtDNA copy number are being considered an important common denominator in several types of human cancers, including CRC [55]. It has been previously reported that oxidative stress, which we found to be increased in these samples [12], may result in increased mitochondrial mass and, therefore, mtDNA copy number in some types of cancers [56]. It is also possible that the mtDNA copy number is increased to compensate for the loss of OXPHOS complexes we observed without necessarily increasing the mitochondrial pool. Interestingly, the mtDNA copy number usually increases with the tumor stage in CRC [56]. In this sense, recent studies are evaluating its use as a potential biomarker for the risk of developing CRC and for patients’ prognosis [57,58]. Considering that metastasis is a multistage process, a better understanding of the mechanisms underlying its development is essential to design new therapeutic strategies. 

## 5. Conclusions

The present work describes key aspects of the energetic metabolic profile in human tumor tissue samples and non-tumor adjacent tissues from stages III and IV of CRC, which reveal the differences in mitochondrial function between a pre-metastatic and a metastatic stage. Taken together, our results showed a shift in the oxidative metabolic profile between the pre-metastatic stage III and the metastatic stage IV of CRC. The stage III tumor tissue showed an oxidant metabolic profile very similar to that of the non-tumor adjacent tissue with a tendency to promote mitochondrial function towards metastasis. Once in the metastatic stage (IV), the oxidative metabolism with aerobic glycolysis would contribute to maintaining the metastatic phenotype, with signs of mitochondrial function impairment in metastatic tumor tissue. This suggests that energy metabolism may influence the process of metastasis in human CRC and could be targeted to prevent the progression of this type of cancer.

## Figures and Tables

**Table 1:** mtDNA quantification in tissues from stage III and IV of human CRC.

CRC	Stage III		Stage IV		
	Non-Tumor Adjacent Tissue	Tumor Tissue	Non-Tumor Adjacent Tissue	Tumor Tissue	
Content of mtDNA	100 ± 7.66	121 ± 2.73 *	93 ± 2.82	118 ± 1.84 *	T

Data are presented as mean ± SEM. ANOVA analysis: two-way (*n* = 11 per group); T: tissue effect; * difference between tumor tissue and non-tumor adjacent tissue.

## Data Availability

The data presented in this study are available on request from the corresponding author (J.O.). The data are not publicly available due to them being human-related data.

## Supplementary Materials

The following are available online at https://www.mdpi.com/article/10.3390/biology11020293/s1, Figure S1: Representative images of the different load controls for Western blot analysis, Figure S2: Comparison between a primary cancer cell line (HT29) and a metastatic cancer cell line (SW620), Figure S3: Data analysis from public gene expression databases, Method S1: Loading controls for Western Blot analysis, Method S2: Cell culture, RT-qPCR, and Western blot analysis. Method S3: Data analysis from public datasets

## References

[1] Benson A.B. (2007). Epidemiology, disease progression, and economic burden of colorectal cancer. J. Manag. Care Pharm..

[2] Lucas A.S., O’Neil B.H., Goldberg R.M. (2011). A Decade of Advances in Cytotoxic Chemotherapy for Metastatic Colorectal Cancer. Clin. Colorectal Cancer.

[3] Sanoff H.K., Sargent D.J., Campbell M.E., Morton R.F., Fuchs C.S., Ramanathan R.K., Williamson S.K., Findlay B.P., Pitot H.C., Goldberg R.M. (2008). Five-Year Data and Prognostic Factor Analysis of Oxaliplatin and Irinotecan Combinations for Advanced Colorectal Cancer: N9741. J. Clin. Oncol..

[4] Guaragnella N., Giannattasio S., Moro L. (2014). Mitochondrial dysfunction in cancer chemoresistance. Biochem. Pharmacol..

[5] Porporato P.E., Ry V., Payen L., Pé Rez-Escuredo J., De Saedeleer C.J., Danhier P., Copetti T., Dhup S., Tardy M., Vazeille T. (2014). A Mitochondrial Switch Promotes Tumor Metastasis. Cell Rep..

[6] Heiden M.G.V., Cantley L.C., Thompson C.B., Mammalian P., Exhibit C., Metabolism A. (2009). Understanding the Warburg Effect: The Metabolic Requirements of Cell Proliferation. Science.

[7] Warburg O. (1956). On the origin of cancer cells. Science.

[8] Moreno-Sánchez R., Rodríguez-Enríquez S., Marín-Hernández A., Saavedra E. (2007). Energy metabolism in tumor cells. FEBS J..

[9] Hanahan D., Weinberg R.A., Pan K.H., Shay J.W., Cohen S.N., Taylor M.B., Clarke N.W., Jayson G.C., Eshleman J.R., Nowak M.A. (2011). Hallmarks of Cancer: The Next Generation. Cell.

[10] Jose C., Bellance N. (2011). Choosing between glycolysis and oxidative phosphorylation: A tumor’s dilemma?. Biochim. Biophys. Acta Bioenerg..

[11] Potter V.R. (1958). The biochemical approach to the cancer problem. Fed. Proc..

[12] Hernández-López R., Torrens-Mas M., Pons D.G., Company M.M., Falcó E., Fernández T., Ibarra de la Rosa J.M., Sastre-Serra J., Oliver J., Roca P. (2018). Non-tumor adjacent tissue of advanced stage from CRC shows activated antioxidant response. Free Radic. Biol. Med..

[13] Bergmeyer H.U., Bergmeyer J., Grassl M. (1983). Methods of Enzymatic Analysis. Vol. 3: Enzymes 1. Oxidoreductases, Transferases.

[14] Chrzanowska-Lightowlers Z.M.A., Turnbull D.M., Lightowlers R.N. (1993). A Microtiter Plate Assay for Cytochrome c Oxidase in Permeabilized Whole Cells. Anal. Biochem..

[15] Ragan C.I., Wilson M.T., Darley-Usmar V.M., Lowe P.N. (1987). Subfractionation of mitochondria and isolation of the proteins of oxidative phosphorylation. Mitochondria A Practical Approach.

[16] Feichtinger R.G., Neureiter D., Skaria T., Wessler S., Cover T.L., Mayr J.A., Zimmermann F.A., Posselt G., Sperl W., Kofler B. (2017). Oxidative Phosphorylation System in Gastric Carcinomas and Gastritis. Oxidative Med. Cell. Longev..

[17] Kumari S., Badana A.K., Murali Mohan G., Shailender G., Malla R.R. (2018). Reactive Oxygen Species: A Key Constituent in Cancer Survival. Biomark. Insights.

[18] Nesci S., Trombetti F., Pagliarani A., Ventrella V., Algieri C., Tioli G., Lenaz G. (2021). Molecular and supramolecular structure of the mitochondrial oxidative phosphorylation system: Implications for pathology. Life.

[19] Galadari S., Rahman A., Pallichankandy S., Thayyullathil F. (2017). Reactive oxygen species and cancer paradox: To promote or to suppress?. Free Radic. Biol. Med..

[20] Valle A., Santandreu F.M., Garcia-Palmer F.J., Roca P., Oliver J. (2008). The serum levels of 17beta-estradiol, progesterone and triiodothyronine correlate with brown adipose tissue thermogenic parameters during aging. Cell. Physiol. Biochem..

[21] Pons D.G., Torrens-Mas M., Nadal-Serrano M., Sastre-Serra J., Roca P., Oliver J. (2015). The presence of Estrogen Receptor β modulates the response of breast cancer cells to therapeutic agents. Int. J. Biochem. Cell Biol..

[22] Luna F., Roca P., Oliver J., Antenucci C.D. (2012). Maximal thermogenic capacity and non-shivering thermogenesis in the South American subterranean rodent Ctenomys talarum. J. Comp. Physiol. B.

[23] Blanquer-Rosselló M.d.M., Hernández-López R., Roca P., Oliver J., Valle A. (2017). Resveratrol induces mitochondrial respiration and apoptosis in SW620 colon cancer cells. Biochim. Biophys. Acta Gen. Subj..

[24] Guevara R., Gianotti M., Roca P., Oliver J. (2011). Age and Sex-Related Changes in Rat Brain Mitochondrial Function. Cell. Physiol. Biochem..

[25] Torrens-Mas M., González-Hedström D., Abrisqueta M., Roca P., Oliver J., Sastre-Serra J. (2017). PGC-1α in melanoma: A key factor for antioxidant response and mitochondrial function. J. Cell. Biochem..

[26] Pons D.G., Nadal-Serrano M., Torrens-Mas M., Oliver J., Roca P. (2016). The Phytoestrogen Genistein Affects Breast Cancer Cells Treatment Depending on the ERα/ERβ Ratio. J. Cell. Biochem..

[27] Santandreu F.M., Valle A., Fernandez de Mattos S., Roca P., Oliver J. (2009). Hydrogen peroxide regulates the mitochondrial content of uncoupling protein 5 in colon cancer cells. Cell. Physiol. Biochem..

[28] Acin-Perez R., Gatti D.L., Bai Y., Manfredi G. (2011). Protein phosphorylation and prevention of cytochrome oxidase inhibition by ATP: Coupled mechanisms of energy metabolism regulation. Cell Metab..

[29] Helling S., Vogt S., Rhiel A., Ramzan R., Wen L., Marcus K., Kadenbach B. (2008). Phosphorylation and kinetics of mammalian cytochrome c oxidase. Mol. Cell. Proteom..

[30] Li Y., Park J.S., Deng J.H., Bai Y. (2006). Cytochrome c oxidase subunit IV is essential for assembly and respiratory function of the enzyme complex. J. Bioenerg. Biomembr..

[31] Chekulayev V., Mado K., Shevchuk I., Koit A., Kaldma A., Klepinin A., Timohhina N., Tepp K., Kandashvili M., Ounpuu L. (2015). Metabolic remodeling in human colorectal cancer and surrounding tissues: Alterations in regulation of mitochondrial respiration and metabolic fluxes. Biochem. Biophys. Rep..

[32] Graziano F., Ruzzo A., Giacomini E., Ricciardi T., Aprile G., Loupakis F., Lorenzini P., Ongaro E., Zoratto F., Catalano V. (2017). Glycolysis gene expression analysis and selective metabolic advantage in the clinical progression of colorectal cancer. Pharm. J..

[33] Sjoblom T., Jones S., Wood L.D., Parsons D.W., Lin J., Barber T.D., Mandelker D., Leary R.J., Ptak J., Silliman N. (2006). The Consensus Coding Sequences of Human Breast and Colorectal Cancers. Science.

[34] Xu W., Yang H., Liu Y., Yang Y., Wang P., Kim S.-H., Ito S., Yang C., Wang P., Xiao M.-T. (2011). Oncometabolite 2-Hydroxyglutarate Is a Competitive Inhibitor of α-Ketoglutarate-Dependent Dioxygenases. Cancer Cell.

[35] Jones A.W.E., Yao Z., Vicencio J.M., Karkucinska-Wieckowska A., Szabadkai G. (2012). PGC-1 family coactivators and cell fate: Roles in cancer, neurodegeneration, cardiovascular disease and retrograde mitochondria–nucleus signalling. Mitochondrion.

[36] Lagouge M., Argmann C., Gerhart-Hines Z., Meziane H., Lerin C., Daussin F., Messadeq N., Milne J., Lambert P., Elliott P. (2006). Resveratrol Improves Mitochondrial Function and Protects against Metabolic Disease by Activating SIRT1 and PGC-1α. Cell.

[37] Wu Z., Puigserver P., Andersson U., Zhang C., Adelmant G., Mootha V., Troy A., Cinti S., Lowell B., Scarpulla R.C. (1999). Mechanisms controlling mitochondrial biogenesis and respiration through the thermogenic coactivator PGC-1. Cell.

[38] Puigserver P., Wu Z., Park C.W., Graves R., Wright M., Spiegelman B.M. (1998). A cold-inducible coactivator of nuclear receptors linked to adaptive thermogenesis. Cell.

[39] Feilchenfeldt J., Bründler M.A., Soravia C., Tötsch M., Meier C.A. (2004). Peroxisome proliferator-activated receptors (PPARs) and associated transcription factors in colon cancer: Reduced expression of PPARgamma-coactivator 1 (PGC-1). Cancer Lett..

[40] Vellinga T.T., De Boer V.C.J., Fatrai S., Van Schelven S., Trumpi K., Verheem A., Snoeren N., Emmink B.L., Koster J., Rinkes I.H.M.B. (2015). SIRT1/PGC1a-Dependent increase in oxidative phosphorylation supports chemotherapy resistance of colon cancer. Clin. Cancer Res..

[41] Cannino G., Di Liegro C.M., Rinaldi A.M. (2007). Nuclear–mitochondrial interaction. Mitochondrion.

[42] Kanki T., Ohgaki K., Gaspari M., Gustafsson C.M., Fukuoh A., Sasaki N., Hamasaki N., Kang D. (2004). Architectural Role of Mitochondrial Transcription Factor A in Maintenance of Human Mitochondrial DNA. Mol. Cell. Biol..

[43] Kaufman B.A., Durisic N., Mativetsky J.M., Costantino S., Hancock M.A., Grutter P., Shoubridge E.A. (2007). The mitochondrial transcription factor TFAM coordinates the assembly of multiple DNA molecules into nucleoid-like structures. Mol. Biol. Cell.

[44] Gabrielson M., Björklund M., Carlson J., Shoshan M. (2014). Expression of mitochondrial regulators PGC1α and TFAM as putative markers of subtype and chemoresistance in epithelial ovarian carcinoma. PLoS ONE.

[45] Piantadosi C.A., Suliman H.B. (2006). Mitochondrial transcription factor A induction by redox activation of nuclear respiratory factor 1. J. Biol. Chem..

[46] Lu B., Lee J., Nie X., Li M., Morozov Y.I., Venkatesh S., Bogenhagen D.F., Temiakov D., Suzuki C.K. (2013). Phosphorylation of Human TFAM in Mitochondria Impairs DNA Binding and Promotes Degradation by the AAA+ Lon Protease. Mol. Cell.

[47] Yoshida Y., Izumi H., Torigoe T., Ishiguchi H., Itoh H., Kang D., Kohno K. (2003). p53 physically interacts with mitochondrial transcription factor A and differentially regulates binding to damaged DNA. Cancer Res..

[48] Jiang X., Wang J. (2019). Down-regulation of TFAM increases the sensitivity of tumour cells to radiation via p53/TIGAR signalling pathway. J. Cell. Mol. Med..

[49] Li H.Y., Lin X.W., Geng S.L., Xu W.H. (2018). TGF-β and BMP signals regulate insect diapause through Smad1-POU-TFAM pathway. Biochim. Biophys. Acta Mol. Cell Res..

[50] Wu K., Ma J., Zhan Y., Liu K., Ye Z., Chen J., Xu K., Huang H., He Y. (2018). Down-Regulation of MicroRNA-214 Contributed to the Enhanced Mitochondrial Transcription Factor A and Inhibited Proliferation of Colorectal Cancer Cells. Cell. Physiol. Biochem..

[51] Channakkar A.S., Singh T., Pattnaik B., Gupta K., Seth P., Adlakha Y.K. (2020). MiRNA-137-mediated modulation of mitochondrial dynamics regulates human neural stem cell fate. Stem Cells.

[52] Jiang J., Yang J., Wang Z., Wu G., Liu F. (2013). TFAM is directly regulated by miR-23b in glioma. Oncol. Rep..

[53] Gaya-Bover A., Hernández-López R., Alorda-Clara M., Ibarra de la Rosa J.M., Falcó E., Fernández T., Company M.M., Torrens-Mas M., Roca P., Oliver J. (2020). Antioxidant enzymes change in different non-metastatic stages in tumoral and peritumoral tissues of colorectal cancer. Int. J. Biochem. Cell Biol..

[54] Feng S., Xiong L., Ji Z., Cheng W., Yang H. (2011). Correlation between increased copy number of mitochondrial DNA and clinicopathological stage in colorectal cancer. Oncol. Lett..

[55] Lee H.-C., Yin P.-H., Lin J.-C., Wu C.-C., Chen C.-Y., Wu C.-W., Chi C.-W., Tam T.-N., Wei Y.-H. (2005). Mitochondrial Genome Instability and mtDNA Depletion in Human Cancers. Ann. N. Y. Acad. Sci..

[56] Lee H.C., Wei Y.H. (2009). Mitochondrial DNA instability and metabolic shift in human cancers. Int. J. Mol. Sci..

[57] Shuwen H., Xi Y., Yuefen P. (2017). Can Mitochondria DNA Provide a Novel Biomarker for Evaluating the Risk and Prognosis of Colorectal Cancer?. Dis. Markers.

[58] Wang Y., He S., Zhu X., Qiao W., Zhang J. (2016). High Copy Number of Mitochondrial DNA Predicts Poor Prognosis in Patients with Advanced Stage Colon Cancer. Int. J. Biol. Markers.

