# Supplementary material for: Mitochondrial Function Differences between Tumor Tissue of Human Metastatic and Premetastatic CRC
Source: Biology (Basel). 2022 Feb 11;11(2):293. doi: 10.3390/biology11020293 (PMC8869310; doi:10.3390/biology11020293)
Supplement: Supplementary file 1 [file biology-11-00293-s001.zip › biology-1548344 - supplementary.pdf]

*Article*

# Mitochondrial Function Differences between Tumor Tissue of Human Metastatic and Premetastatic CRC

**Reyniel Hernández-López [1,2], Margalida Torrens-Mas [1,3], Daniel G. Pons [1,2], Maria M. Company [2,4] Esther Falcó [2,5], Teresa Fernández [2,5], Javier M. Ibarra de la Rosa [2,5], Pilar Roca [1,2,6], Jordi Oliver [1,2,6,*] and Jorge Sastre-Serra [1,2,6]**

[1] Grupo Multidisciplinar de Oncología Traslacional, Institut Universitari d´Investigació en Ciències de la Salut (IUNICS), Universitat de les Illes Balears, E-07122 Palma de Mallorca, Illes Balears, Spain; r.hernandez@uib.es (R.H-L.); margalida.torrens@ssib.es (M.T-M.); d.pons@uib.es (D.G.P.); pilar.roca@uib.es (P.R.); jorge.sastre@uib.es (J.S-S.)

[2] Instituto de Investigación Sanitaria de las Islas Baleares (IdISBa), Hospital Universitario Son Espases, edificio S, E-07120 Palma de Mallorca, Illes Balears, Spain; mmcompany@hsll.es (M.M.C.); efalco@hsll.es (E.F.); tfernandez@hsll.es (T.F.); jibarra@hsll.es (J.M.I.R.)

[3] Translational Research in Aging and Longevity (TRIAL) Group, Instituto de Investigación Sanitaria de las Islas Baleares (IdISBa), E-07120, Palma, Spain

[4] Servicio de Anatomía Patológica, Clinica Rotger, 07012 Palma de Mallorca, Islas Baleares, Spain

[5] Servicio de Oncología, Hospital Son Llatzer, 07198 Palma de Mallorca, Illes Balears, Spain

[6] Ciber Fisiopatología Obesidad y Nutrición (CB06/03) Instituto Salud Carlos III, E-28029, Madrid, Spain

* Correspondence: jordi.oliver@uib.es

**Supplementary data**

*Supplemental Method S1—Loading controls for Western Blot analysis*

Different loading controls for the Western Blot analyses were used. The TGX-Stain Free system from BioRad was used for all gels. First, every gel was loaded and run as stated in the methods section. Once the electrophoresis was completed, each gel was exposed to UV light in the Chemidoc XRS densitometer to visualize the proteins in each lane. After the transfer of the gel to a membrane as explained in the methods section, each gel was visualized again under UV light to analyze the equal transfer to the membrane. These controls, as indicated by the manufacturer's instruction, allow the normalization of the bands to total protein of each lane, which therefore eliminate the need for probing for housekeeping proteins. Nevertheless, a Ponceau staining of the membrane after the transfer was also performed for each blot. All three images were quantified using the Quantity One Software. If no significant changes were found, the blot proceeded as explained in the methods section. This method has been published and validated before in [1,2].

*Supplemental Figure S1*

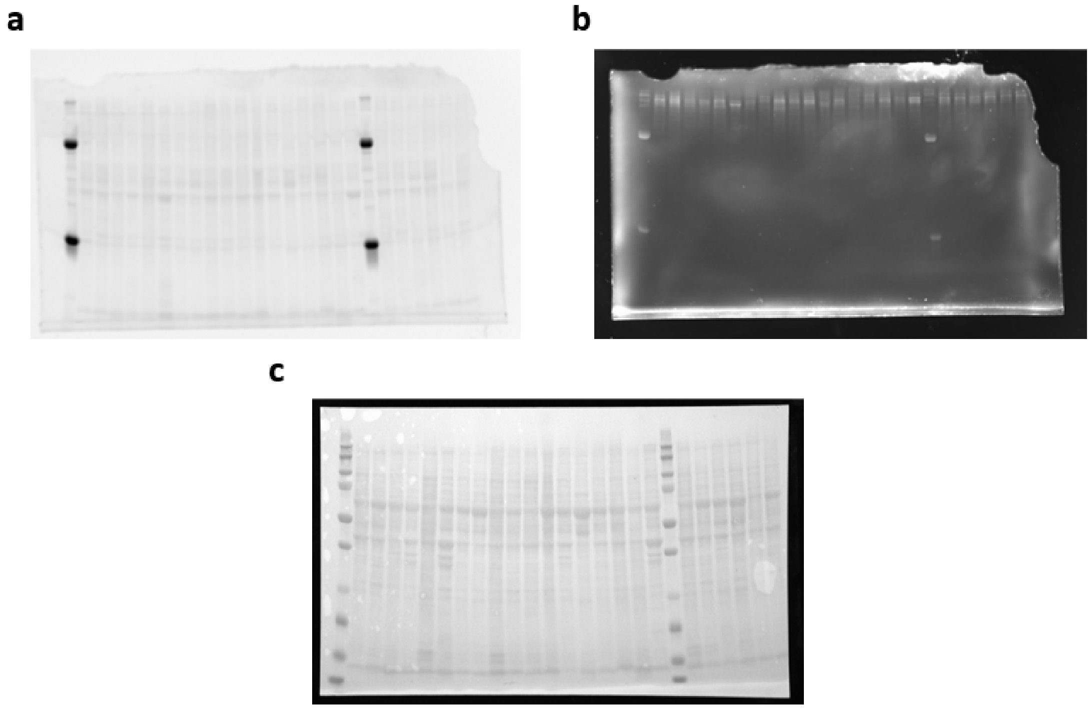


**Figure S1.** Representative images of the different load controls for Western Blot analysis. (**a**) Image of the gel after running it; (**b**) Image of the gel after transfer to membrane; (**c**) Image of membrane Ponceau Staining.

*Supplemental Method S2—Cell culture and RT-qPCR and Western Blot analysis*

HT-29 and SW620 cell lines were obtained from ATCC American Type Culture Collection (ATCC; Manassas, VA, USA) and were maintained in DMEM media supplemented with 10% (v/v) fetal bovine serum (FBS) and 1% (v/v) penicillin and streptomycin at 37 °C and 5% CO2. Once the culture reached confluence, cells were harvested with TRI Reagent® (Sigma-Aldrich) and RNA was isolated following manufacturer's instructions. RNA was reverse transcribed, and the resulting cDNA was used to perform PCR using SYBR Green Technology. The primers used were as follows: *PPARGC1A* FW 5′-TCATGCCGTGGTAAGTACCA-3′, RV 5′-GTGCAAAGTTCCCTCTCTGC-3′; *COXIV* FW 5′-AACGAGTGGAAGACGGTTGT-3′, RV 5′-AACGAGTGGAAGACGGTTGT-3; *18S* 5'-GGACACGGACAGGATTGACA-3', RV 5'-ACCCACGGAATCGAGAAAGA-3'. Furthermore, cells were also harvested with RIPA buffer 50 mM Tris-HCl pH 7.5, 150 mM NaCl, 0.1% SDS, 0.5% deoxycholate, 1% Triton X-100, 1 mM EDTA, and protease and

phosphatase inhibitors) for Western Blot analysis. Protein content quantification and Western Blot analysis were performed as explained in the main manuscript.

*Supplemental Figure S2*

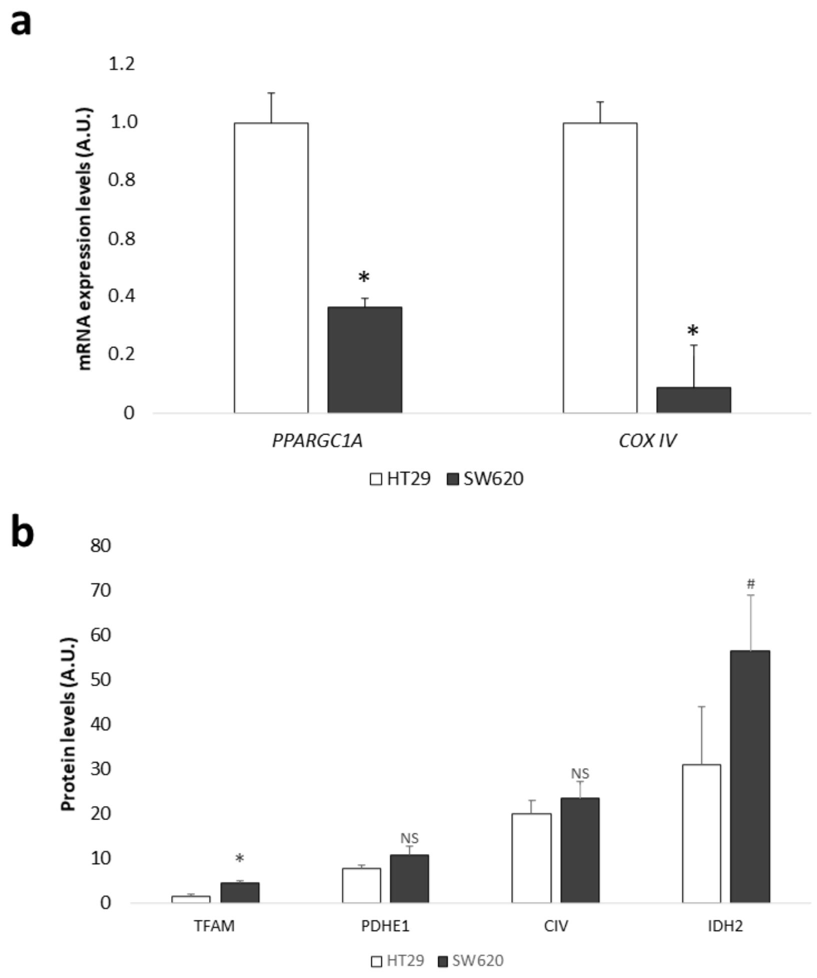


**Figure S2.** Comparison between a primary cancer cell line (HT29) and a metastatic cancer cell line (SW620). (**a**) mRNA levels of *PPARGC1A* and *COX IV* are decreased in the metastatic cell line compared to the primary cell line. (**b**) Protein levels of TFAM are increased in the metastatic line, while PDHE1 and OXPHOS complex IV levels do not show a significant increase. Finally, IDH2 levels were increased in the metastatic cell line. *$p \leq 0.05$.

*Supplemental Method S3—Data analysis from public datasets*

Several public databases were queried to check for differences in gene expression in different samples. TNMplot tool [3] was used to analyze the expression of COX4I1 gene in normal colon tissue, colon tumoral tissue, and metastatic colon tissue. Furthermore, GEO datasets were also used to compare the expression of several genes in stage III CRC and stage IV CRC (accession numbers GSE27913 and GSE21510), and in primary and metastatic CRC cell lines (accession numbers GSE2509 and GSE1323).

*Supplemental Figure S3*

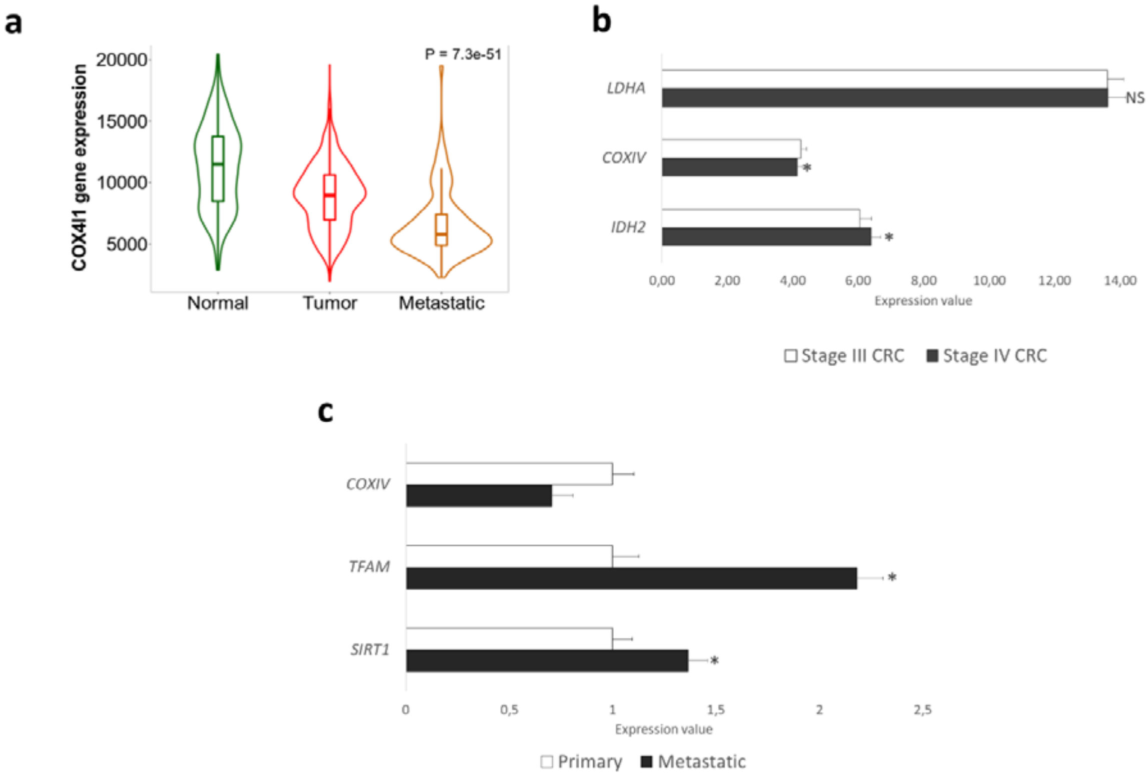


**Figure S3.** Data analysis from public gene expression databases. (**a**) Comparison of COX4I1 expression in normal colon tissue, colon tumoral tissue, and colon metastatic tissue. Significant decrease in the COXIV gene expression levels in the metastatic tissue compared to the tumor tissue is observed (FC = 0.74; Dunn test = 3,41E-41). These data have been obtained from the TNMplot tool, available at https://www.tnmplot.com/. (**b**) Comparison of gene expression of *LDHA*, *COXIV*, and *IDH2* in stage III CRC and stage IV CRC human samples. These data have been obtained from the GEO dataset with accession number GSE27913 and are similar to the ones obtained from the dataset with accession number GSE21510. (**c**) Comparison of expression levels of *SIRT1*, *TFAM*, and *COXIV* between two cell lines derived from the same patient, SW480 corresponding to a primary tumor, and SW620, corresponding to a metastatic tumor. Data available at the GEO datasets with accession numbers GSE2509 and GSE1323. *$p \leq 0.05$.

## Supplemental references


1. Hernandez-Lopez et al. Non-tumor adjacent tissue of advanced stage from CRC shows activated antioxidant response. *Free Radic Biol Med*. 2018, 126:249-258. doi: 10.1016/j.freeradbiomed.2018.08.021.
2. Gaya-Bover et al. Antioxidant enzymes change in different non-metastatic stages in tumoral and peritumoral tissues of colorectal cancer. *Int J Biochem Cell Biol*. 2020, 120:105698. doi: 10.1016/j.biocel.2020.105698.
3. Bartha Á, Győrffy B. TNMplot.com: A Web Tool for the Comparison of Gene Expression in Normal, Tumor and Metastatic Tissues. *International Journal of Molecular Sciences*. 2021, 22(5):2622. https://doi.org/10.3390/ijms22052622.
