# Supplementary figures and images for: Mitochondrial Function Differences between Tumor Tissue of Human Metastatic and Premetastatic CRC
Source: Biology (Basel). 2022 Feb 11;11(2):293. doi: 10.3390/biology11020293 (PMC8869310; doi:10.3390/biology11020293)

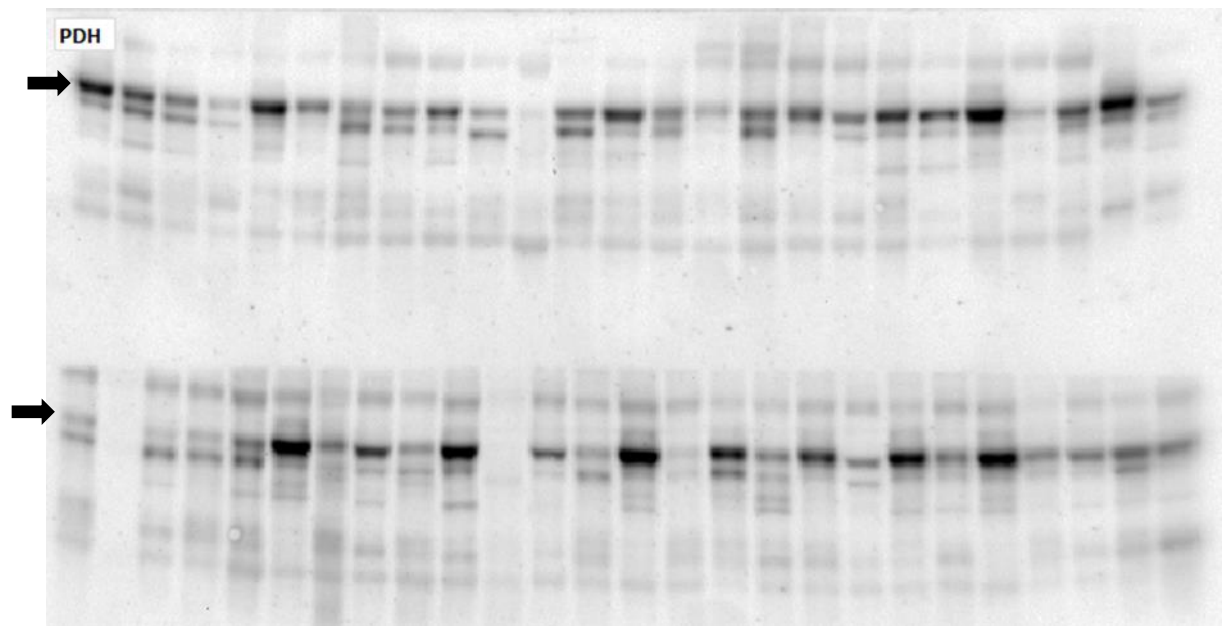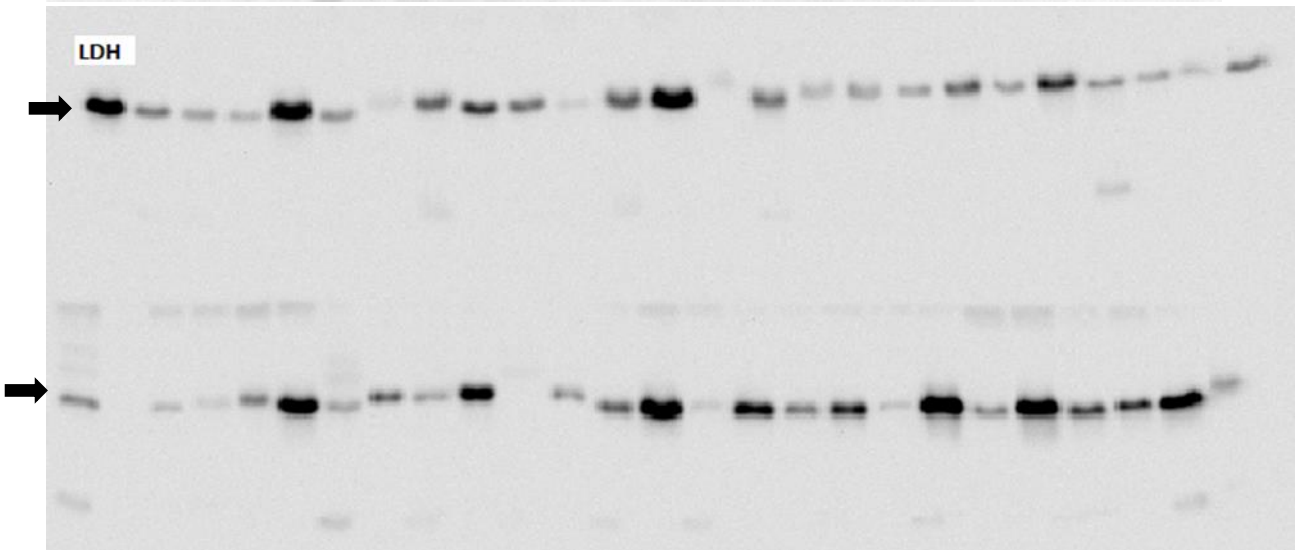

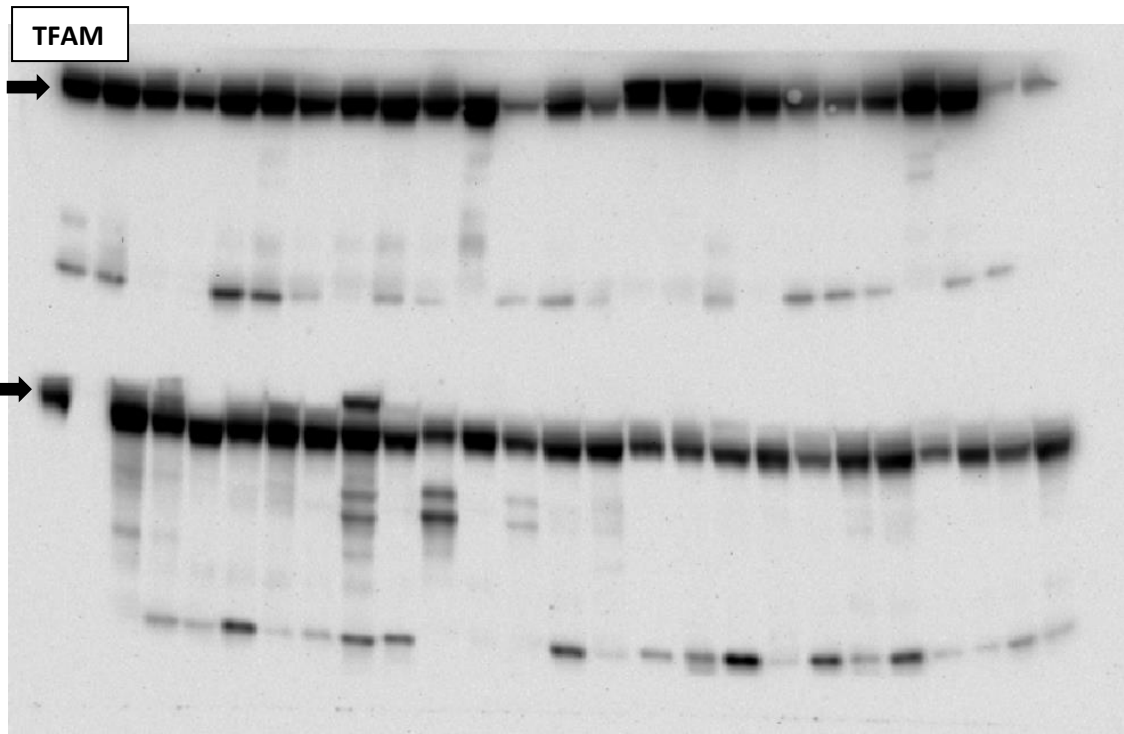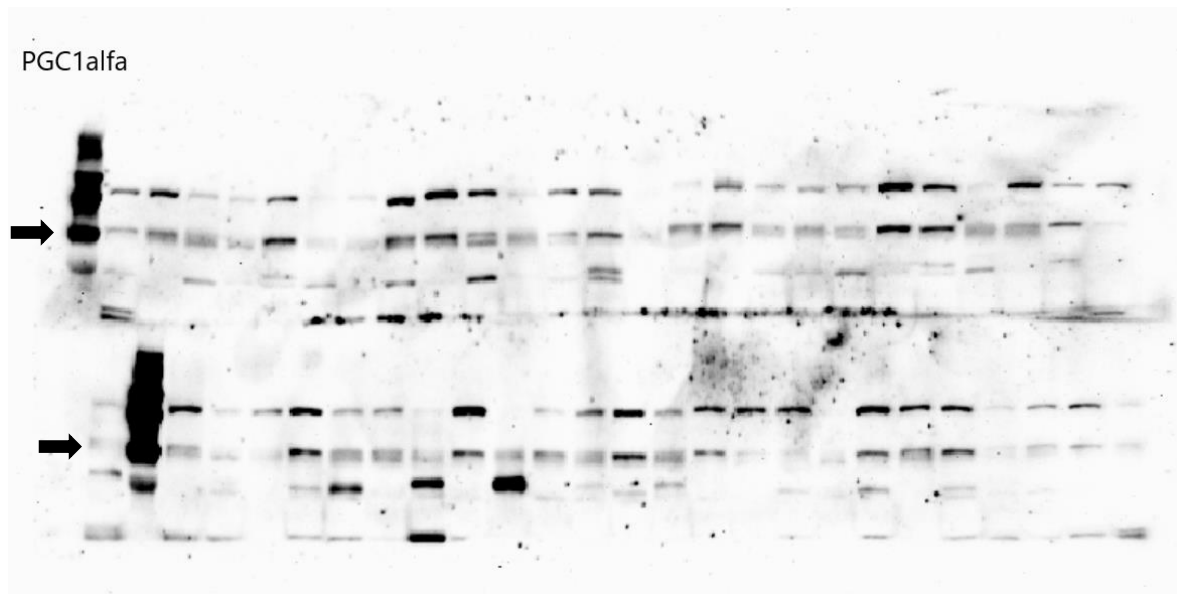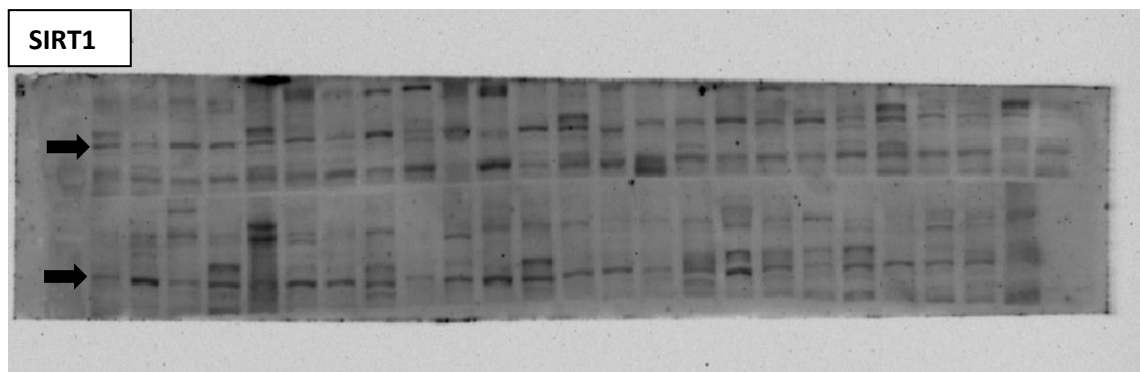

IDH2

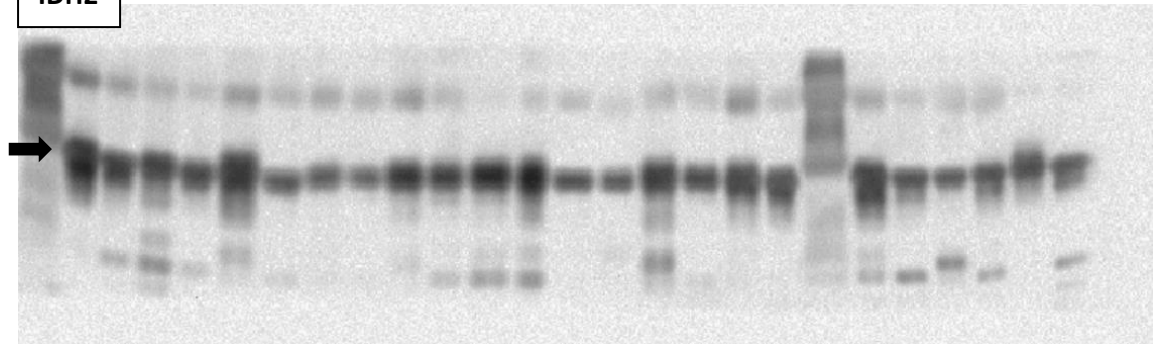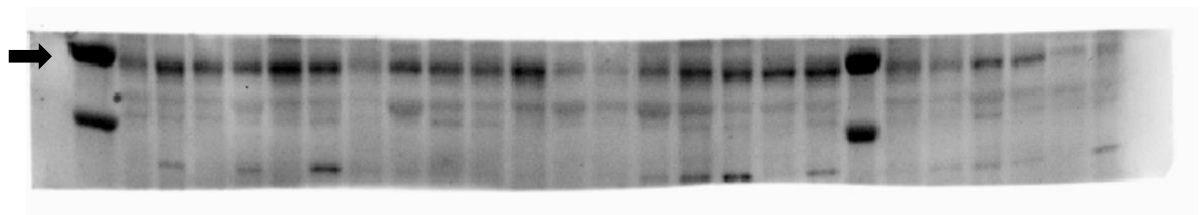

OXPHOS

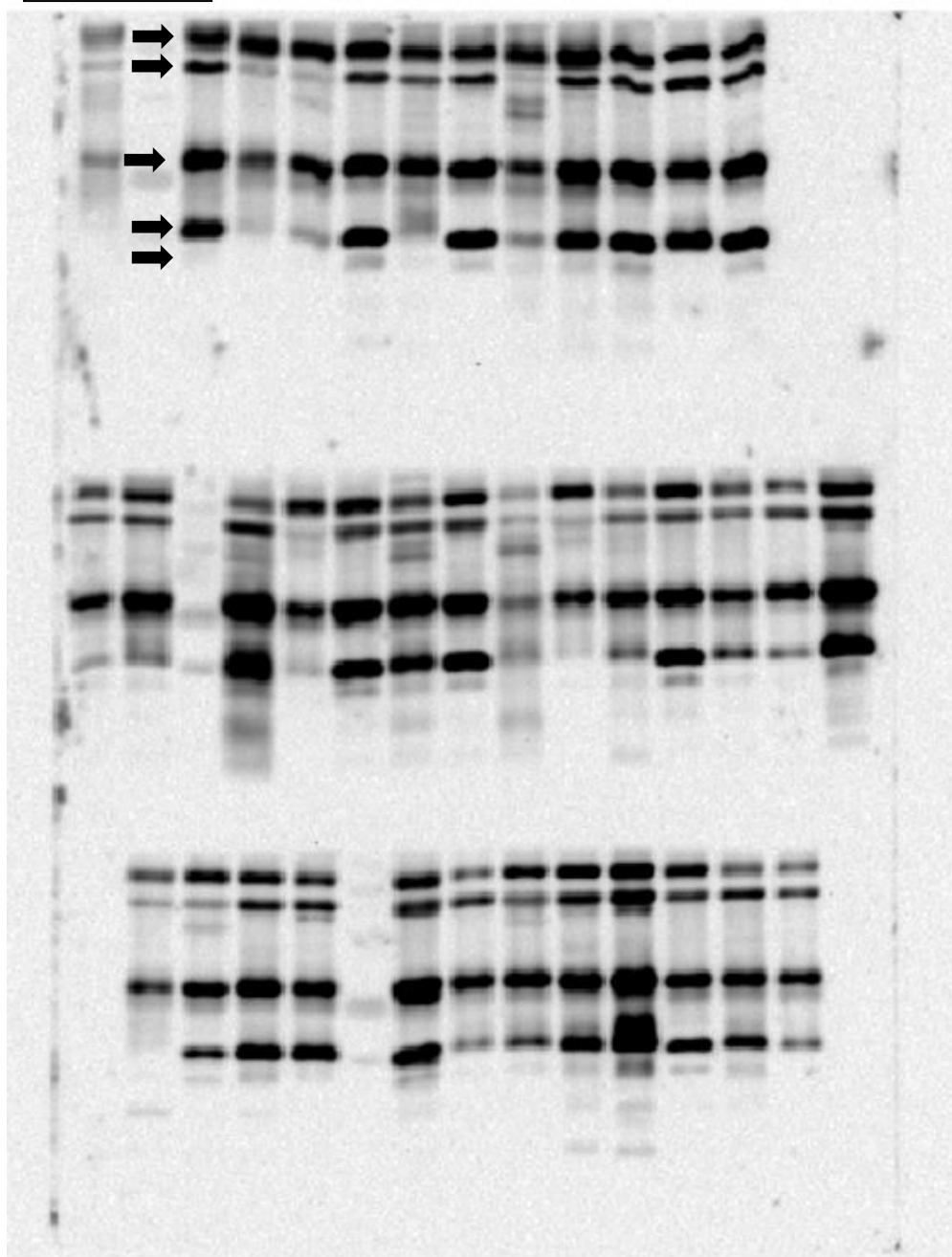

Supplement: Supplementary file 1 [file biology-11-00293-s001.zip › biology-1548344 - original images.pdf]
